# Knowledge and practice of the Global Initiative for Asthma report among community pharmacists in a Nigerian State

**DOI:** 10.11604/pamj.2020.37.83.18897

**Published:** 2020-09-23

**Authors:** Kosisochi Chinwendu Amorha, Kingsley Emeka Idoko, Mathew Jegbefume Okonta, Chinwe Victoria Ukwe

**Affiliations:** 1 Department of Clinical Pharmacy and Pharmacy Management, Faculty of Pharmaceutical Sciences, University of Nigeria, Nsukka, Enugu State, Nigeria

**Keywords:** Asthma, community pharmacists, GINA, knowledge, practice

## Abstract

**Introduction:**

few studies have been conducted to evaluate pharmacists´ knowledge and practice of the asthma guidelines. The Global Initiative for Asthma (GINA) report was developed to reduce practice variability and to improve the quality of asthma care. This study aimed to assess the knowledge and practice of the GINA report among community pharmacists in a Nigerian State.

**Methods:**

this cross-sectional survey was conducted among community pharmacists in Enugu State, Nigeria (May to July, 2018). Data were collected with a 39-item structured self-administered questionnaire and analyzed using the IBM SPSS Version 21.0. Descriptive statistics were used to summarize data. Inferential statistics utilized the Pearson Chi-Square test where applicable, with statistical significance set at P < 0.05.

**Results:**

a total of 89 community pharmacists in Enugu State participated in the study (76.7% participation rate). More than half of them were less than 40 years old (60.7%), male (59.6%) and only had the Bachelor of Pharmacy (B.Pharm) degree (83.1%). About a tenth of the community pharmacists (10.1%) reported that they stock the peak flow meter. Few of them (2.2%) utilized the Asthma Control Test™ in their practice. After categorization, less than half of the community pharmacists had good knowledge of asthma (34.8%) and demonstrated good practice of the GINA report (11.2%).

**Conclusion:**

the community pharmacists had poor knowledge of asthma and demonstrated poor practice of the GINA report. With adequate knowledge of the guidelines, community pharmacists can assist patients with making informed decisions and proffer appropriate recommendations to physicians.

## Introduction

Asthma has been recognized as a public health problem that negatively affects patients and their caregivers [1, 2]. It reduces the quality of life of patients and escalates their health care costs [3-5]. About 339 million people have asthma worldwide [6]. In Nigeria, poor standard of living is a bottleneck in the management of asthma [6]. Guidelines for clinical practice assist health care professionals and patients to make informed decisions about diseases [7]. The Global Initiative for Asthma (GINA) was instituted to intensify asthma awareness among healthcare professionals and members of the community in a bid to improve asthma prevention and management [1]. Despite the availability of guidelines for the care of patients with asthma, practice patterns have been reported to differ among health professionals based on extent of specialization in respiratory medicine and patient factors [8]. The existing variations might also stem from access to medicines. Some of the recommendations in clinical practice guidelines may not be applicable in resource-limited settings [9].

In Nigeria, for instance, inhaled corticosteroids (ICS) are not commonly available such that patients are prescribed ICS in combination with long-acting β2-agonists (LABA) when they can benefit from ICS alone [6]. Specific country needs might necessitate a national asthma management guideline. The first edition of the Guideline for Asthma Management in Nigeria was launched on 2^nd^ May 2017 by the Nigeria Thoracic Society [6]. Before this, the practice of asthma management in Nigeria was generally based on adaptations from the GINA reports. Few studies have researched the utilization of the asthma guidelines among pharmacists. Community pharmacists have continuously evolving roles and are well-positioned in the community to render asthma-related services, including referrals to physicians and specialists, without the need for appointments before consultations [10]. As of the time of conducting this study, the GINA reports were readily accessible by health professionals, including pharmacists and there was no concern whether information on the availability of a new national guideline was efficiently disseminated. This study sought to assess the knowledge and practice of the GINA report among community pharmacists in Enugu State, Nigeria. It may reveal the gaps in community pharmacists´ involvement with asthma management, especially as regards making recommendations to prescribers and asthma patients. By understanding the specific areas of deficit, better interventions can be designed.

## Methods

**Study design and sample population:** this was a cross-sectional survey conducted among community pharmacists in Enugu State, South-East, Nigeria, over a period of three months (May to July 2018). When this study was conducted, there were 116 registered community pharmacists in Enugu State. The eligibility criteria included all registered community pharmacists who worked in Enugu State that were willing to participate.

**Data collection:** the study instrument was a 38-item structured self-administered questionnaire divided into sections on: demographic information; frequency of involvement with asthma care; knowledge of asthma based on the GINA report; practice of the GINA report and barriers to adherence to the GINA report. The questionnaire was adapted and modified from previous studies [8, 11, 12]. Modifications were made after the questionnaire was content-validated by clinical pharmacists in the University of Nigeria Nsukka, for easy comprehension and readability. It was pre-tested among community pharmacists that were excluded from the study. The survey was conducted by the researcher who met with each community pharmacist. The objectives of the study were explained and oral consent for participation was sought before the questionnaires were administered. The community pharmacists filled the questionnaire in their pharmacies without the consultation of any reference material. Confidentiality was maintained as neither the names of the community pharmacists nor pharmacies were requested for.

**Data analysis:** data were analyzed using the IBM SPSS Version 21.0 (IBM Corp, Version 21.0, Armonk, NY, USA). Descriptive statistics, such as mean ± standard deviation, were used to summarize data. Inferential statistics utilized the Pearson Chi-square test where applicable, with statistical significance set at P < 0.05.

**Ethical consideration:** this study was conducted based on the approved protocol from the Health Research and Ethics Board of the University of Nigeria Teaching Hospital (UNTH), Ituku-Ozalla, Enugu State.

## Results

A total of eighty-nine (89) questionnaires were completed and returned, representing a participation rate of 76.7% (89/116). More than half of the community pharmacists were less than 40 years old (60.7%), male (59.6%) and only had the Bachelor of Pharmacy (B.Pharm) degree (83.1%). Close to a quarter of them (23.6%) had more than 10 years community pharmacy experience (Table 1). Only about a tenth of the community pharmacists (10.1%) claimed to stock the peak flow meter. Few of them (2.2%) utilized the Asthma Control Test^™^in their practice. A higher proportion of the pharmacists (43.8%) reported that asthma patients visit their community pharmacies only few times per year. In addition, a higher proportion of the community pharmacists (40.4%) reported filling prescriptions for/recommending asthma medications few times per year Many of the community pharmacists (87.6%) knew that coughing, wheezing and recurrent chest tightness are the major symptoms of asthma. However, only about half of them (53.9%) knew that the use of more than one canister of short-acting inhaled β2-agonists per month indicates inadequate disease control (Table 2). The total knowledge score was obtained and categorized with the cut-off point as the median score such that those with scores above the median were classified as having good knowledge. After categorization, only 34.8% of the community pharmacists had good knowledge of asthma based on the GINA report.

**Table 1:** demographic of the community pharmacists in the survey, n = 89

Variables	n (%)
**Age (in years)**	****
20 – 29	26 (29.2)
30 – 39	28 (31.5)
40 – 49	23 (25.8)
50 – 59	1 (1.1)
>60	11 (12.4)
**Gender**	****
Male	53 (59.6)
Female	36 (40.4)
**Number of years after graduation**	****
<5	22 (24.7)
5 – 10	33 (37.1)
11 – 20	19 (21.3)
>20	15 (16.9)
**Community pharmacy experience (in years)**	****
<5	48 (53.9)
5 – 10	20 (22.5)
11 – 20	14 (15.7)
>20	7 (7.9)
**Highest qualification**	****
M.Sc/M.Pharm	7 (7.9)
MPH	3 (3.4)
FPCPharm	5 (5.6)
Ph. D	0 (0.0)
B.Pharm	74 (83.1)

**Table 2:** knowledge of asthma based on the GINA report, n = 89

Statements (correct answer)	n (%)
The use of more than one canister of short-acting inhaled β2-agonists per month indicates inadequate disease control (true)	48 (53.9)
Inhaled corticosteroids act as direct bronchodilators (false)	42 (47.2)
Salmeterol xinafoate is an example of an inhaled steroid (false)	42 (48.3)
Cough which is worse at night, wheezing and recurrent chest tightness are suggestive of asthma (true)	78 (87.6)
The use of long-acting inhaled β2-agonists can help many asthmatics discontinue steroids (false)	26 (29.2)
Inhaled anticholinergic agents are not the most indicated drugs for long-term control of asthma (true)	55 (61.8)
For asthma patients without symptoms between attacks, short-acting inhaled β2-agonists, as needed, are usually sufficient (true)	52 (58.4)
Patient education in the management of asthma cannot improve inhaler technique (false)	81 (91.0)

Although many of the community pharmacists claimed to know the major symptoms suggestive of asthma, only 7.9% provided the correct answer to a scenario of a 40-year-old with coughing and wheezing at nights, overusing salbutamol inhaler with little relief. None of them correctly stated that asthma patients should always be with their reliever inhaler and use when necessary even if they have had no symptoms of asthma in less than a year (Table 3). The total practice score was obtained and categorized with the cut-off point as the median score such that those with scores above the median were classified as demonstrating good practice. After categorization, only about a tenth (11.2%) of the community pharmacists demonstrated good practice of the GINA report. About half of the community pharmacists (55.1%) agreed that lack of a counselling room could be a barrier to adhering to the GINA report. More than half of the community pharmacists agreed that lack of asthma records (85.4%) and lack of training (74.2%) are barriers to adhering to the GINA report (Table 4). There was no statistically significant association between the demographic variables and the community pharmacists´ knowledge and practice of the GINA report (Table 5).

**Table 3:** practice of the GINA report, n = 89

Scenario (correct answer)	n (%)
A 40 y/o male with cough and wheezing at nights; overuses salbutamol inhaler with little relief (prescribe a single ingredient inhaled steroid)	7 (7.9)
2. A 35 y/o female with poor asthma control; on regular fluticasone 250mcg twice daily and salbutamol inhaler when necessary (Prescribe a long acting beta agonist (LABA) or combination of inhaled steroid/LABA)	3 (3.4)
3. A 72 y/o female with symptoms upon exertion; on regular budesonide 160mcg/formoterol fumarate 4.5mcg once daily and salbutamol inhaler when necessary (Increase dose of the combination inhaled steroid/LABA to twice daily)	3 (3.4)
A 27 y/o male collecting salbutamol inhaler prescription; has had no symptoms in the last 9 months (no action. He should use the salbutamol inhaler only when necessary or as needed)	0 (0.0)
A 62 y/o female on fluticasone 250mcg/salmeterol 50mcg twice daily and both salbutamol and ipratropium for relief (removal of second reliever)	1 (1.1)
A 65 y/o male has a cold, with symptoms of a lot of trouble breathing and trouble sleeping. He is currently on 160mcg/formoterol fumarate 4.5mcg twice daily; salbutamol inhaler for relief, when necessary; montelukast 10mg once daily in the evening (prescribe a short course of oral steroid)	1 (1.1)
A 43 y/o female has not had to use salbutamol inhaler for a very long time; feels well. She has been on fluticasone 100mcg/salmeterol 50mcg twice daily (remove LABA)	1 (1.1)

**Table 4:** barriers to adherence to the GINA report, n = 89

Variables	SD	D	A	SA	Mean (SDv)
**Institutional barriers**	****	****	****	****	****
Lack of interviewing place/counselling room	7 (7.9)	33 (37.1)	24 (27.0)	25 (28.1)	2.75 (0.96)
Lack of peak flow meters	5 (5.6)	22 (24.7)	38 (42.7)	24 (27.0)	2.91 (0.86)
Lack of required medications	7 (7.9)	25 (28.1)	35 (39.3)	22 (24.7)	2.81 (0.90)
Lack of asthma action plan	1 (1.1)	10 (11.2)	51 (57.3)	27 (30.3)	3.17 (0.66)
Lack of asthma records	0 (0.0)	13 (14.6)	47 (52.8)	29 (32.6)	3.18 (0.67)
Lack of multi-disciplinary approach	0 (0.0)	17 (19.1)	48 (53.9)	24 (27.0)	3.08 (0.68)
Defective referral system	1 (1.1)	11 (12.4)	42 (49.2)	35 (39.3)	3.25 (0.71)
**Barriers related to pharmacists**	****	****	****	****	****
Lack of training	5 (5.6)	18 (20.2)	34 (38.2)	32 (36.0)	3.04 (0.89)
Lack of knowledge	2 (2.2)	24 (27.0)	38 (42.7)	25 (28.1)	2.97 (0.80)
Time constraints	0 (0.0)	24 (27.0)	35 (39.3)	30 (33.7)	3.07 (0.78)
**Barriers related to patients**	****	****	****	****	****
Not complying with management	0 (0.0)	2 (2.2)	31 (34.8)	56 (62.9)	3.61 (0.54)
Not adhering to follow-up schedules	0 (0.0)	0 (0.0)	36 (40.4)	53 (59.6)	3.60 (0.49)
Hiding the disease	1 (1.1)	11 (12.4)	33 (37.1)	44 (49.4)	3.35 (0.74)

SD = strongly disagree (coded as 1); D = disagree (coded as 2); A = agree (coded as 3); SA = strongly agree (coded as 4); SDv = standard deviation

**Table 5:** association between the demographic variables and community pharmacists’ knowledge and practice of the GINA report, n = 89

Variables	Poor knowledge	Good knowledge	Total	χ^2^	P-value	Poor practice	Good practice	Total	χ^2^	P-value
**Age (in years)**	****	****	****	**1.716**	**0.788**	****	****	****	**2.051**	**0.726**
20 – 29	16 (27.6)	10 (32.3)	26 (29.2)			22 (27.8)	4 (40.0)	26 (29.2)		
30 – 39	17 (29.3)	11 (35.5)	28 (31.5)			25 (31.6)	3 (30.0)	28 (31.5)		
40 – 49	17 (29.3)	6 (19.4)	23 (25.8)			20 (25.3)	3 (30.0)	23 (25.8)		
50 – 59	1 (1.7)	0 (0.0)	1 (1.1)			1 (1. 3)	0 (0.0)	1 (1.1)		
>60	7 (12.1)	4 (12.9)	11 (12.4)			11 (13.9)	0 (0.0)	11 (12.4)		
**Gender**	****	****	****	**1.325**	**0.250**	****	****	****	**0.001**	**0.975**
Male	32 (55.2)	21 (67.1)	53 (59.6)			47 (59.5)	6 (60.0)	53 (59.6)		
Female	26 (44.8)	10 (32.3)	36 (40.4)			32 (40.5)	4 (40.0)	36 (40.4)		
**Number of years after graduation (in years)**	****	****	****	**2.808**	**0.422**	****	****	****	**4.014**	**0.260**
<5	12 (20.7)	10 (32.3)	22 (24.7)			19 (24.1)	3 (30.0)	22 ( 24.7)		
5 – 10	24 (41.4)	9 (29.0)	33 (37.1)			30 (38.0)	3 (30.0)	33 (37.1)		
11 – 20	11 (19.0)	8 (25.8)	19 (21.3)			15 (19.0)	4 (40.0)	19 ( 21.3)		
>20	11 (19.0)	4 (12.9)	15 (16.9)			15 (19.0)	0 (0.0)	15 (16.9)		
**Community pharmacy experience (in years)**	****	****	****	**5.231**	**0.156**	****	****	****	**1.333**	**0.721**
<5	31 (53.4)	17 (54.8)	48 (53.9)			43 (54.1)	5 (50.0)	48 (53.9)		
5 – 10	15 (25.9)	5 (16.1)	20 (22.5)			17 (21.5)	3 (30.0)	20 (22.5)		
11 – 20	6 (10.3)	8 (25.8)	14 (15.7)			12 (15.9)	2 (20.0)	14 (15.7)		
>20	6 (10.3)	1 (3.2)	7 (7.9)			7 (8.9)	0 (0.0)	7 (7.9)		

P <0.05 shows statistical significance

## Discussion

More than half of the community pharmacists that participated were less than 40 years old. Most of them were male and had no additional qualification. Close to a quarter had more than 10 years community pharmacy experience. Only about a tenth of the participants claimed to stock the peak flow meter. Few of them utilized the Asthma Control Test^™^in their practice. A high proportion of the pharmacists claimed that they had few asthma patients per year. Although many of the community pharmacists knew the major symptoms suggestive of asthma, less than a tenth provided the correct answer to a scenario of a 40-year-old with coughing and wheezing at nights, overusing salbutamol inhaler with little relief. After categorization, less than half of the community pharmacists had good knowledge of asthma based on the GINA report and only about a tenth demonstrated good practice of the GINA report. Only about a tenth of the community pharmacists claimed to stock peak flow meters. This might be due to their minimal involvement with asthma care, rarity of prescriptions for the peak flow meter or poor knowledge of the use of the device [13]. Similarly, in a study in Alberta, Canada, most of the community pharmacies had no peak flow meter in stock and the greatest number any of the pharmacies had dispensed was six in a year [14]. Peak flow meters are also poorly stocked in Nigerian tertiary hospitals [15]. These might provide evidence for the extent to which peak flow meters are used to objectively measure lung function in Nigeria. The GINA guidelines advocate the use of symptom control and lung function to monitor asthma [1].

Few of the community pharmacists utilized the Asthma Control Test^™^(ACT) in their practice. This might be due to poor awareness on the ACT. In a research conducted among the primary care physicians in Vietnam it was found out that only 25% used the asthma control test to assess the asthma of their patients [11]. The ACT is a simple, quick test and reliable way to assess asthma [16]. In a busy practice with limited time and resources, the ACT provides an easy method for assessing asthma control with or without the lung function testing [16]. It is quite worrisome that the community pharmacists were neither great at assessing symptom control nor lung function testing with the peak flow meter. Another Nigerian study revealed that the overall knowledge of asthma control measurement tools among doctors was poor with the highest score among pulmonologists, doctors in pulmonary specialty and those that attended Continuing Medical Education (CME) in less than six months [17]. Our study revealed that overall, less than half of the community pharmacists were able to provide correct answers to the knowledge-based questions on asthma. This might be due to deficiency in the knowledge of the report. A poor understanding of the GINA report by physicians managing asthma patients has also been reported in a tertiary hospital in Nigeria [18].

A study conducted among physicians in South-west Nigeria observed gaps regarding the use of the GINA report. [19]. High level of experience in asthma specialty can present as good knowledge of the asthma guidelines [8,18]. Most community pharmacists are generalists in practice. The community pharmacists in our study fared poorly in the practice-based scenarios. If they are to be relevant in the management of asthma, then further training is required. This corroborates with the findings of a scenario-based Australian study where the pharmacists´ recommendations did not always agree with the guidelines [12]. More than half of the community pharmacists agreed that lack of knowledge and lack of training could be barriers to adherence to the GINA report. If the community pharmacists are unaware of the GINA reprt or have poor knowledge of its contents, it would most likely present as poor practice as they would not know when they go astray. There should be increased awareness of the Guideline for Asthma Management in Nigeria which was recently launched by the Nigeria Thoracic Society.

Community pharmacists need to be well-grounded to make informed decisions considering that they are the custodians of drugs and asthma patients might present to their premises to fill prescriptions, seek counsel or for other health-related needs. If pharmacists are well informed, they would be able to dispense specific information on asthma drugs to prescribers, as well as recognize the critical role of the physician in diagnosis and review [12]. These would translate to making prompt referrals when necessary and channels for collaboration among health professionals. This study has some limitations. The findings might not be generalized to other states in Nigeria. In addition, community pharmacists are majorly generalists in practice, thus their knowledge of specific guidelines or reports might not be comparable to physicians who are specialists in pulmonology. Furthermore, the scenarios were intended to depict the clinical presentations of real-life patients. However, it is possible that the information provided to the pharmacists about the patients in the scenarios were limited and the pharmacists might have made more informed decisions if they were capable of having in-depth discussions with the patients or utilizing their reference books as could occur in practice. There should be increased awareness of the Guideline for Asthma Management in Nigeria, launched by the Nigeria Thoracic Society, among community pharmacists. This would ensure uniformity in recommendations and practice. Future studies can research into the nationwide utilization of the asthma guidelines or reports among community pharmacists.

## Conclusion

The community pharmacists had poor knowledge of asthma and demonstrated poor practice of the Global Initiative for Asthma (GINA) report. Participation in asthma care was minimal. Community pharmacists should continually update their knowledge of the asthma guidelines or reports to help asthma patients and other health professionals make informed decisions, especially those pertaining to drugs. There is need for constant training and re-training of pharmacists by seminars, workshops, conferences and academic programmes.

### What is known about this topic

- Asthma is a public health problem that negatively affects patients and their caregivers;
- Guidelines for the management of diseases are developed to reduce practice variability and improve the quality of care.

### What this study adds

- Community pharmacists have pivotal roles to play in asthma management but participation in asthma care is minimal;
- Community pharmacists should continually update their knowledge of the asthma guidelines to help asthma patients and other health professionals make informed decisions.
